# Effect of 0.02% and 0.01% atropine on astigmatism: a two-year clinical trial

**DOI:** 10.1186/s12886-022-02385-z

**Published:** 2022-04-07

**Authors:** Ming Wang, Can Cui, Yao Sui, Shi-Ao Yu, Jing-Xue Ma, Ai-Cun Fu

**Affiliations:** 1 The First Affiliated Hospital of Zhengzhou University, No. 1 Jianshe road, Zhengzhou, 450000 China; 2 The Second Affiliated Hospital of Zhengzhou University, Zhengzhou, 450000 China; 3 The Second Hospital of Hebei Medical University, Heping West Road No. 215, Shi Jiazhuang, 050000 China

**Keywords:** Low-concentration, Atropine, Myopia, Children, Astigmatism

## Abstract

**Background:**

To evaluate the effects of 0.02% and 0.01% atropine eye drops on ocular and corneal astigmatism over 2 years.

**Methods:**

A prospective clinic-controlled trail. The cohort study assessed 400 myopic children and divided them into three groups: 138 and 142 children were randomized to use either 0.02% or 0.01% atropine eye drops, respectively. They wore single-vision (SV) spectacles, with one drop of atropine applied to both eyes once nightly. Control children (*n* = 120) only wore SV spectacles. Spherical equivalent refractive errors (SER) and corneal curvature were measured every 4 months. The SER and corneal curvature were assessed by cycloplegic autorefraction and IOLMaster. Ocular and corneal astigmatism were calculated by Thibos vector analysis and then split into its power vector components, J0 (with-the-rule astigmatism) and J45 (oblique).

**Results:**

After 2 years, the ocular astigmatism increased by -0.38 ± 0.29 D, -0.47 ± 0.38 D, -0.41 ± 0.35 D in the 0.02%, 0.01% atropine groups and control group, respectively (*p* = 0.15). The corresponding corneal astigmatism increased by -0.20 ± 0.34 D, -0.28 ± 0.35 D and -0.26 ± 0.26 D (*p* = 0.18). The ocular astigmatism J0 increased by 0.19 ± 0.28 D, 0.22 ± 0.36 D, 0.18 ± 0.31 D in the 0.02% atropine, 0.01% atropine and control groups, respectively (*p* = 0.65). The corresponding corneal astigmatism J0 increased by -0.05 ± 0.34 D, -0.11 ± 0.37 D and -0.13 ± 0.30 D (*p* = 0.23). There was a small but significant increase in ocular astigmatism (including J0) (all *P* < 0.05), but there were no changes in the ocular astigmatism J45 and corneal astigmatism (including J0 and J45) in the three groups over time (all *p* > 0.05). However, there were no significant differences in the changes in ocular astigmatism (including J0) among the three groups.

**Conclusions:**

Treatment with 0.02% and 0.01% atropine had no clinically significant effect on ocular and corneal astigmatism over 2 years.

**Trial registration:**

The First Affiliated Hospital of Zhengzhou University, ChiCTR-IPD-16008844. Registered 14/07/2016.

## Background

The prevalence of myopia and high myopia has increased worldwide in recent decades because of the changes in lifestyle and behavior [1]. Recently, several studies, including ours, showed that low-concentration atropine could safely and effectively control the progression of myopia in children and have slight rebound effects after withdrawal [2–14]. Among these studies, the safety research included no best-corrected visual acuity loss, no change in intraocular pressure [6], no effect on the quality and quantity of tear film [14], visual sensitivity [10, 11], and stereoscopic and vision-related quality of life [4, 10]. Meanwhile, there was no evidence of retinal and optic nerve toxicity or photodamage with low-concentration atropine in the literature [2, 7, 9, 10, 12]. The most common ocular symptoms due to the use of low-concentration atropine are photophobia and near-vision blur [5, 13, 15]. However, these symptoms are mild and tolerable and do not affect studies and daily activities. Additionally, allergy and inflammation [8] are important side effects, but they will resolve spontaneously after the withdrawal of the low-concentration atropine.

Astigmatism is another important parameter for evaluating the safety of low-concentration atropine in children. To date, only one study [16] reported that 0.05%, 0.025%, and 0.01% atropine did not affect corneal astigmatism in children in a one-year follow-up, however, they did not perform a vector analysis of astigmatism. Astigmatism is a vector with both value and direction. In this two-year clinical trial, vector analysis was used to observe the effect of 0.02% and 0.01% atropine eye drops on ocular astigmatism and corneal astigmatism in myopic children.

## Methods

Details of the methodology and study design have been published elsewhere and are briefly described here. Four hundred right eyes of Chinese children with myopia (Han nationality) who visited the First Affiliated Hospital of Zhengzhou University were recruited into this cohort study between July 2016 and June 2018. The inclusion criteria were: 6–14 years of age, spherical equivalent refractive errors (SER) from -1.25 to -6.00 D, astigmatism < 2.0 D, anisometropia < 1.0 D, monocular best-corrected visual acuity of 16/20 or better, intraocular pressures between 10 and 21 mmHg, and absence of other eye diseases and past surgery. Exclusion criteria were: previous use of atropine, pirenzepine, rigid gas-permeable, and orthokeratology lenses to control myopia progression, and failure to comply with the study’s visiting schedule.

At the randomization visit, eligible participants were given the option of atropine or no atropine, per the human ethics committee of requirements, and the atropine groups were subsequently assigned in a double-blinded and randomized manner to either 0.01% or 0.02%. This study conformed to the tenets of the Declaration of Helsinki. The possible risks were fully explained before treatment commenced. The experimental drug (1% atropine eye drops; Eye and ENT Hospital Affiliated to Fudan University) was diluted with saline (also with ethyl hydroxybenzoate) to either 0.01% or 0.02% concentrations on a clean bench; eye drops dispensed in 3 mL sealed bottle, stored at 15–25 °C room temperature, and discarded after opening the bottle for 1 month.

Children were reassessed at the 1-month monitoring visit after starting atropine and then at 4, 8, 12, and 24 months. At every visit, all examinations were performed by the same clinician who was blinded to the experimental group of each subject. The children in the control group were prescribed full-correction single-vision (SV) spectacles with the highest positive/least negative power consistent with optimum visual acuity for constant wear. Both experimental groups wore the SV spectacles prescribed under the same protocol as the control group and received one drop of atropine eye drops into both eyes once nightly before sleeping.

Corneal curvatures were evaluated using a non-contact partial coherence interferometer (IOLMaster; Carl Zeiss, Germany). On each occasion, five consecutive measurements were taken, and their means were used for analysis. Cycloplegic autorefraction was performed after instilling four drops of compound tropicamide eye drops (0.5% tropicamide and 0.5% neo-synephrine) (Santen, Japan) administered 10 min apart in each patient’s eye. Ten minutes after instilling the fourth drop, three autorefraction measurements were taken (Topcon RM 8000A, CA), and the mean was obtained. The degree of myopia was expressed as SER. Ocular astigmatism and corneal astigmatism were calculated using Thibos [17] vector analysis. Thibos uses a Fourier conversion formula to convert refraction to M (SER), J0, and J45. Ocular and corneal astigmatism were converted into rectangular vector coordinates as follows:

1 $$\mathrm{M }=\mathrm{ S }+\mathrm{ C}/2$$

2 $$J0 = (-C/2) cos2\alpha$$

3 $$J45 = (-C/2) sin2\alpha$$

S represents a spherical lens. C (minimum–maximum keratometry) stands for the column lens, which denotes the amount of astigmatism at axis α. C is denoted by the negative values. α represents the axial position of the negative column lens. J0 and J45 are the horizontal or vertical and oblique (45° or 135°) components of astigmatism, respectively. A positive J0 indicates that the negative column axis is at 180°, and a negative J0 indicates that the negative column axis is at 90°. A positive J45 indicates that the negative column axis is at 45° and a negative J45 indicates that the negative column axis is at 135°. The ocular astigmatic components were denoted as OJ0 and OJ45, and the corneal astigmatic components were denoted as CJ0 and CJ45. The changes in astigmatism were evaluated by comparing ocular and corneal astigmatism (all including J0 and J45) before and after atropine use by Thibos vector analysis [17, 18].

Continuous baseline variables were expressed as mean ± SD and evaluated using analysis of variance. Categorical variables, such as sex, were expressed as percentages (%) and evaluated using the Chi-squared test. A generalized additive mixed model was used to estimate the longitudinal trend with time (at baseline, 4, 8,12, and 24 months) for dependent variables (SER, corneal curvature, ocular astigmatism (J0 and J45), and corneal astigmatism (J0 and J45) and differences in the rate of change between the three groups. The change represents the slope for each treatment group of dependent variables over time, and the change difference represents the difference in the slope of dependent variables over time between groups. Statistical significance was set at *P* < 0.05. All statistical analyses were performed using Empower (www.empowerstats. com; X & Y Solutions, Boston, MA, USA) and R (http://www.R-project.org).

## Results

**Fig. 1:**
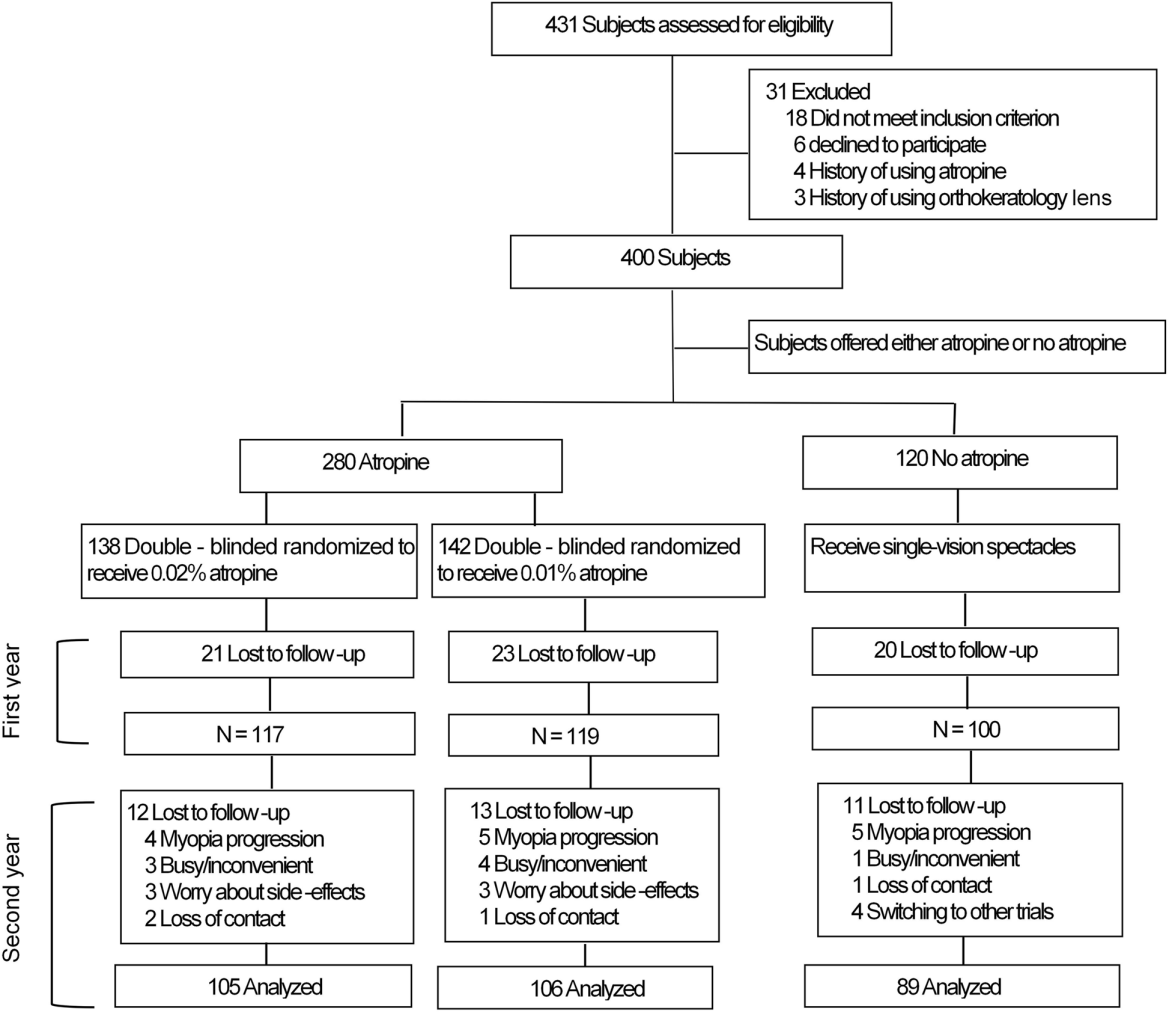
Subject recruitment and randomization flowchart

A total of 400 children were enrolled in this cohort study. There were 138, 142, and 120 children in the 0.02% atropine, 0.01% atropine, and control groups, respectively (Fig. 1). No differences were found in age, sex, intraocular pressure, corneal curvature, SER, ocular astigmatism (J0 and J45), or corneal astigmatism (J0 and J45) between the groups (Table 1). Of the 400 children enrolled, 336 successfully completed the 24-month follow-up examinations. Sixty-four subjects (16%) dropped out, including 21 (15.2%), 23 (16.1%) and 20 (16.6%) in the 0.02% atropine, 0.01% atropine, and control groups, respectively. There were no significant differences in baseline parameters between the dropout subjects and those who completed the study (*p* > 0.05).

**Table 1:** Baseline characteristics of study participants who completed 2 years versus those who have not

	Completed 2 years (*N* = 300)				Not completed 2 years (*N* = 100)		
Variables	0.02% atropine *N* = 105	0.01% atropine *N* = 106	control group *N* = 89		0.02% atropine *N* = 33	0.01% atropine *N* = 36	control group *N* = 31
	Mean ± SD	Mean ± SD	Mean ± SD	*p* value	Mean ± SD	Mean ± SD	Mean ± SD
Age (year)	9.6 ± 1.8	9.4 ± 1.7	9.3 ± 1.4	0.43	9.3 ± 2.1	9.2 ± 2.5	9.6 ± 2.3
Sex (male, n and %)	55 (52.4%)	55 (51.9%)	47 (52.8%)		18 (54.5%)	20 (55.6%)	15 (48.4%)
Spherical equivalent refractive errors (D)	-2.81 ± 1.47	-2.76 ± 1.56	-2.66 ± 1.39	0.78	-2.70 ± 1.79	-2.65 ± 1.88	-2.72 ± 1.75
Intraocular pressure (mmHg)	15.9 ± 3.1	16.9 ± 2.8	17.0 ± 3.0	0.06	15.9 ± 3.1	16.9 ± 2.8	17.0 ± 3.0
Ocular astigmatism (D)							
J0	0.16 ± 0.25	0.19 ± 0.27	0.17 ± 0.23	0.68	0.18 ± 0.25	0.17 ± 0.25	0.20 ± 0.24
J45	0.00 ± 0.15	-0.01 ± 0.09	0.00 ± 0.06	0.65	-0.01 ± 0.12	-0.02 ± 0.09	-0.01 ± 0.08
Total	-0.43 ± 0.49	-0.42 ± 0.54	-0.34 ± 0.47	0.41	-0.41 ± 0.45	-0.41 ± 0.51	-0.36 ± 0.46
Corneal curvature (D)							
Flattest	42.79 ± 1.50	42.81 ± 1.33	42.90 ± 1.09	0.83	42.81 ± 1.56	42.83 ± 1.44	42.94 ± 1.32
Steepest	43.98 ± 1.61	43.98 ± 1.45	44.02 ± 1.21	0.98	43.94 ± 1.65	43.99 ± 1.58	44.08 ± 1.46
Corneal astigmatism (D)							
J0	-0.53 ± 0.26	-0.58 ± 0.30	-0.54 ± 0.21	0.35	-0.60 ± 0.28	-0.62 ± 0.33	-0.59 ± 0.19
J45	0.04 ± 0.21	0.06 ± 0.18	0.02 ± 0.13	0.30	0.04 ± 0.20	0.05 ± 0.16	0.04 ± 0.15
Total	-1.15 ± 0.53	-1.22 ± 0.61	-1.12 ± 0.40	0.39	-1.18 ± 0.55	-1.20 ± 0.60	-1.13 ± 0.40

**Fig. 2:**
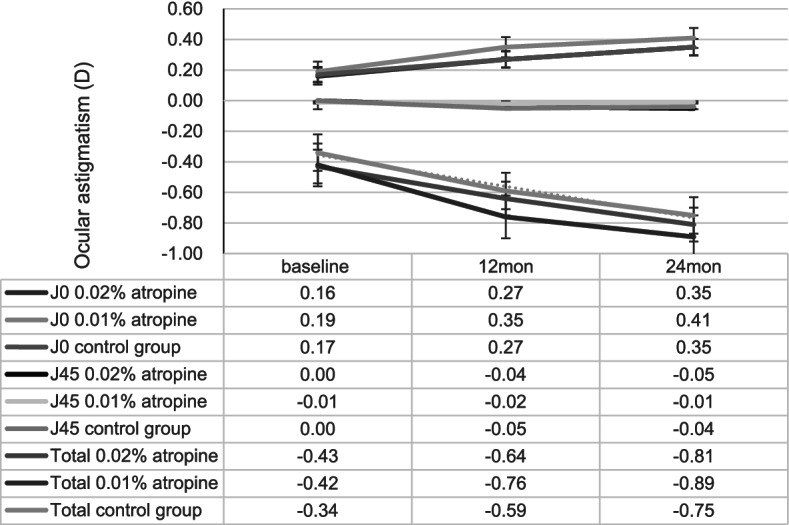
Measurement of ocular astigmatism (J0, J45, and total) over time

At the end of 2 years, ocular astigmatism changes were -0.38 ± 0.29 D, -0.47 ± 0.38 D, -0.41 ± 0.35 D and ocular astigmatism J0 changes were 0.19 ± 0.28 D, 0.22 ± 0.36 D, 0.18 ± 0.31 D in the 0.02%, 0.01% atropine and control groups, respectively. There was a small but significant increase in ocular astigmatism (including J0) (all *p* < 0.05) but no change in J45 (all *p* > 0.05; Fig. 2 and Table 2) in the three groups. However, there were no significant differences in the change in ocular astigmatism (including J0) among the three groups.

**Table 2:** Change and change difference of ocular astigmatism in three groups over 2 years period

Mean (95% CI)										
	0.02% atropine		0.01% atropine		Control group		Change difference between-group			
Variables	Baseline	24 months change	Baseline	24 months change	Baseline	24 months change	0.02% vs. 0.01% atropine	*P* -value	0.01% atropine vs. control group	*P* -value
Ocular astigmatism (J0)	0.16 (0.13 to0.19)	0.008 ^**a**^ (0.006 to 0.01)	0.19 (0.14 to 0.24)	0.009 ^**a**^ (0.005 to 0.013)	0.17 (0.08 to 0.26)	0.008 ^**a**^ (0.002 to 0.014)	-0.001 (-0.003 to 0.001)	0.35	0.001 (-0.001 to 0.003)	0.47
Ocular astigmatism (J45)	0.00 (-0.01 to 0.00)	-0.002 (-0.004 to 0.001)	-0.01 (-0.04 to 0.02)	0 (-0.001 to 0.001)	0.00 (-0.03 to 0.03)	-0.002 (-0.005 to 0.001)	-0.002 (-0.006 to 0.002)	0.10	0.002 (-0.001 to 0.005)	0.24
Total ocular astigmatism	-0.43 (-0.64 to -0.22)	-0.016^**a**^ (-0.024 to -0.008)	-0.42 (-0.60 to -0.24)	-0.02 ^**a**^ (-0.03 to -0.01)	-0.34 (-0.52 to -0.16)	-0.017 ^**a**^ (-0.024 to -0.01)	0.004 (-0.008 to 0.016)	0.26	-0.003 (-0.009 to 0.003)	0.62

*CI* confidence interval. Change: represents the slope of ocular astigmatism (J0, J45, and total) over time for the three groups. Change difference represents the difference in ocular astigmatism (J0, J45, and total) over time between the two groups. ^**a**^Represents: Changes were significantly different. A generalized additive mixed model was used to estimate the longitudinal trend from baseline to 24 months

**Fig.3:**
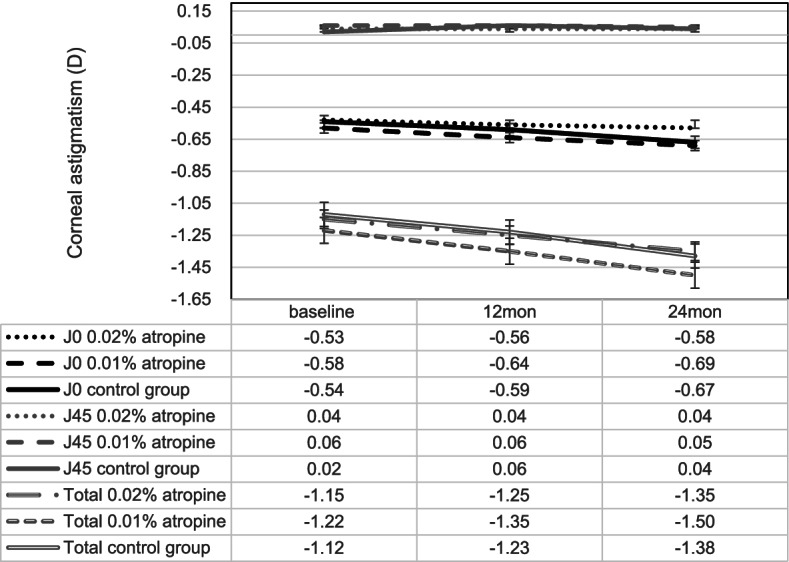
Measurement of corneal astigmatism (J0, J45, and total) over time

At the end of 2 years, corneal astigmatism (minimum–maximum keratometry) changes were -0.20 ± 0.34 D, -0.28 ± 0.35 D, -0.26 ± 0.26 D and corneal astigmatism J0 changes were -0.05 ± 0.30 D, -0.11 ± 0.32 D, -0.13 ± 0.30 D in the 0.02%, 0.01% atropine and control groups, respectively. There was no significant increase in corneal astigmatism (including J0 and J45) and no significant differences in the change in corneal astigmatism (including J0 and J45) among the three groups (all *p* > 0.05, Fig. 3 and Table 3).

**Table 3:** Change and change difference of corneal astigmatism in three groups over 2 years period

Mean (95% CI)										
	0.02% atropine		0.01% atropine		Control group		Change difference between-group			
Variables	Baseline	24 months change	Baseline	24 months change	Baseline	24 months change	0.02% vs. 0.01% atropine	*P* -value	0.01% atropine vs. control group	*P* -value
Corneal astigmatism (J0)	-0.53 (-0.61 to -0.45)	-0.002 (-0.004 to 0.001)	-0.58 (-0.75 to -0.41)	-0.005 (-0.02 to 0.01)	-0.54 (-0.84 to -0.24)	-0.005 (-0.011 to 0.001)	0.006 (-0.002 to 0.014)	0.28	0 (-0.004 to 0.004)	0.75
Corneal astigmatism (J45)	0.04 (0.01 to 0.07)	0 (-0.002 to 0.002)	0.06 (0.02 to 0.10)	0 (–0.001 to 0.001)	0.02 (0 to 0.04)	0.001 (-0.003 to 0.005)	0 (-0.006 to 0.0007)	0.78	-0.001 (-0.005 to 0.003)	0.48
Total corneal astigmatism	-1.15 (-1.45 to -0.85)	-0.008 (-0.017 to 0.001)	-1.22 (-1.32 to -1.12)	-0.012 (-0.026 to 0.002)	-1.12 (-1.4 to -0.82)	-0.011 (-0.03 to 0.008)	0.004 (-0.009 to 0.017)	0.37	-0.001 (-0.006 to 0.004)	0.75

*CI* confidence interval Change: represents the slope of corneal astigmatism (J0, J45, and total) over time for the three groups. Change difference: represents the difference in corneal astigmatism (J0, J45, and total) over time between the two groups. A generalized additive mixed model was used to estimate the longitudinal trend from baseline to 24 months

## Discussion

Our 2-year clinical study showed that ocular astigmatism (including J0) increased to the same extent, but there were no changes in ocular astigmatism J45 and corneal astigmatism (including J0 and J45) for children with myopia among the 0.02% and 0.01% atropine and control groups. Treatment with 0.02% and 0.01% atropine for two years had no clinically significant effects on ocular and corneal astigmatism after vector analysis.

In recent years, several studies have shown that low-concentration atropine can safely and effectively control myopia progression in children, but the influence of low-concentration atropine on astigmatism has rarely been reported. Presently, only one study in Hong Kong [16] randomly assigned 438 myopic children to 0.05% atropine, 0.025% atropine, 0.01% atropine, or placebo in a ratio of 1:1:1:1, and found no differences in corneal astigmatism at various atropine concentrations compared with placebo over 1 year. However, the changes in ocular and corneal astigmatism did not result in vector decomposition. Astigmatism is a vector that has both magnitude and direction. The vector characteristics of astigmatism were easily ignored in the past, but in recent years, many clinicians have paid great attention to it in clinical research [19–21]. Chia et al. [22] found that dropping 1% high concentration atropine did not affect ocular or corneal astigmatism by vector analysis. The eyes of 400 children with myopia were randomly given 1% atropine or placebo daily for 2 years. They were divided into 1% atropine and control groups, and the increase in ocular and corneal astigmatism were similar in both groups after 2 years, which indicates that the change in astigmatism was associated with natural growth and had nothing to do with atropine. Chia et al. [22] also found that the J0 of ocular and corneal astigmatism in the 1% atropine group significantly increased compared to that in the control group during drug drops, but the 1% atropine group changes were consistent with the control group after drug withdrawal, which was slightly different from the results of current study. In current study, the changes in ocular astigmatism (including J0) and corneal astigmatism (including J0) in the atropine and control groups were consistent when using drug drops. The astigmatism was consistent or greater in the atropine group than in the control group during atropine administration, possibly due to the different concentrations of atropine used in the Chia [22] and current studies. Studies have found that the increase in astigmatism is probably due to the flattening and motorial lens during cycloplegia, which is caused by axis tilt around the horizontal axis [23, 24]. Meanwhile, Ye et al. [25] found that the lens became flat and thin, and the lens power decreased after receiving 1% atropine drops, but the thickness and power of the lens did not change after using 0.01% atropine for 1 week. We hypothesized that the less the cycloplegia caused by lower atropine concentrations, the smaller the astigmatism changes. In other words, there was minimal flattening of the lens and no increase in astigmatism when the atropine concentration was low enough (0.01%).

Studies have found that astigmatism is associated with the occurrence and progression of myopia [26–28]. Tong et al. [26] conducted a study on children in Singapore and found that astigmatism was associated with high myopia, with faster progression in children with myopia than in non-myopic children. Meanwhile, studies [27, 28] found that an increase in astigmatism could lead to the occurrence and progression of myopia. In current study, astigmatism changed at the same rate in the 0.02% and 0.01% atropine and control groups, indicating that the change in astigmatism is physiological, that is, the administration of low concentrations atropine had nothing to do with the change of astigmatism.

The study was designed as a randomized controlled trial; however, the proposal from our human ethics committee mandated that at the randomization visit, subjects were to be offered either atropine or no atropine and double-blinded randomization to be carried out only for the two active arms of the trial. However, all subjects were enrolled at the same time, and the baseline data were consistent. The next step will be to adopt a randomized controlled study to verify the current results. In addition, this study only evaluated the effects of low concentration atropine eye drops on ocular and corneal astigmatisms at the level of 0.01% and 0.02%. Studies [13, 16] showed that 0.05% atropine provide a greater efficacy than 0.01%, with still minimal side-effects. Meanwhile, a higher concentration atropine of 1% [22] still does not affect astigmatism would suggest that 0.05% will also have little effect on it. Thus, further studies should be carried out to evaluate the effects of a little higher concentration of low concentrations of atropine such as 0.05% on astigmatism. Moreover, the change in the trend of astigmatism after the withdrawal of atropine was not observed, which is under study.

## Conclusions

0.02% and 0.01% atropine had no clinically significant effect on ocular and corneal astigmatism over 2 years. Considering astigmatism, 0.02%, and 0.01% atropine is safe for myopic children.

## Data Availability

The datasets analyzed in the current study are available from the corresponding author for reasonable requests.

## Abbreviations

SV: Single-Vision
SER: Spherical Equivalent Refractive errors
WTR: With-the-rule
